# Head lice infestation and the role of some cognitive‐behavioral factors in its spread and prevention among adolescent girls: A cross‐sectional study in Northwest Iran

**DOI:** 10.1002/hsr2.1679

**Published:** 2023-11-01

**Authors:** Towhid Babazadeh, Khalil Maleki Chollou, Sanaz Abedi‐Nerbin, Salar Abedi‐Nerbin, Farzaneh Shahnavaz‐Yoshanluie, Soheila Ranjbaran

**Affiliations:** ^1^ Department of Public Health Sarab Faculty of Medical Sciences Sarab Iran; ^2^ Department of Nursing Sarab Faculty of Medical Sciences Sarab Iran; ^3^ Education Organization of Sarab Sarab Iran; ^4^ Education Organization of Ajabshir Ajabshir Iran

**Keywords:** adolescent, cognitive‐behavioral factors, head lice, health promoting behaviors, Iran

## Abstract

**Background and Aims:**

Head lice is a public health problem of worldwide distribution, particularly among school children and girls. Head lice infestation (HLI) can lead to negative social and psychological outcomes such as distress and anxiety in children and their families. Hence, the present study aimed to investigate the role of cognitive‐behavioral factors in its spread and prevention among adolescent girls.

**Methods:**

The cross‐sectional study was conducted among 276 school‐aged adolescent girls from September 2022 to January 2023 in Herris, a city located in Northwest Iran. A multistage cluster random sample was used to recruit adolescent girls in secondary schools. Two schools were randomly selected from five secondary schools. Then, students in each school were randomly selected from a school list. Data were collected using a valid and reliable questionnaire.

**Results:**

The high protective behaviors were significantly associated with the number of family members (*ß* = 0.158; *p* value = 0.012). An additional 21.8% of the variation in preventive behaviors was explained by cognitive factors as predictor variables (*p* value > 0.05). Perceived collective family efficacy, perceived barriers, perceived self‐efficacy, and response efficacy were predictors of head lice preventive behaviors, respectively. Among all variables, perceived collective family efficacy was the strongest predictor.

**Conclusions:**

The findings of current research support the determinants of the cognitive‐behavioral factors in the spread and prevention of HLI. It is better to involve these factors in school‐based educational programs by policymakers and healthcare providers.

## 1 INTRODUCTION

Head lice is a public health problem of worldwide distribution, particularly among school children^1^ and girls are more likely to get head lice than boys.^1^, ^2^, ^3^, ^4^ The prevalence of head lice was 20.4% among Iranian female adolescents.^5^ In the other studies, the prevalence of head lice has been 23.38%,^6^ and 26.3%^7^ in Iran.

Head lice infestation (HLI) can lead to negative social and psychological outcomes such as distress, anxiety in children and their families, social stigma, isolation, blaming in friends groups, impose extra costs, absence from school, and failure in academic performance.^2^, ^8^, ^9^, ^10^, ^11^, ^12^

Some studies have shown that the factors affecting HLI include demographic and socioeconomic factors, lifestyle‑related factors, number of people sharing the room (family size), accessibility to soaps/shampoos, sharing of bed, towels/soaps and combs, bathing, not combing hair, hair longitude and condition, going to the hairdresser, low level of family's knowledge about head lice, its transmission, and treatment, school type (Government/private).^1^, ^13^, ^14^, ^15^, ^16^

Due to the prevalence of head lice in schools and adolescent girls, it seems that some factors affecting this health problem are unknown. According to the study by Nezhadali et al., Cognitive and behavioral factors determined 21% of the variance in predictive behaviors of head lice.^17^ To prevent, screen for, or control unhealthy behavior or condition, these factors: perceived susceptibility, perceived severity, perceived barriers, and self‐efficacy can be affected. When people consider themselves susceptible to a disease, they believe that condition or disease would have critical subsequences, then they believe that the method available to them would help reduce their perceived susceptibility or severity of the condition or disease, and they believe in the benefits of performing behavior outweigh barriers (or costs) of behavior, Eventually, they are likely to perform a behavior that they believe will decrease their risks of the condition or disease.^18^ According to Bandura, self‐efficacy is defined as “the conviction that one can successfully execute the behavior required to produce the outcomes” (Confidence in one's ability to take action).^18^, ^19^ That is to say, people must consider themselves competent (self‐efficient) to overcome barriers of behavior.^18^ Also, response efficacy and perceived collective family efficacy have the main role to perform health‐promoting behaviors.^20^

Perceived collective family efficacy is a supportive factor during crises and stressful situations such as afflicted diseases for children.^21^ “Bandura sustains that the strength of families, communities, organizations, social institutions, and even nations depends partly on people's sense of collective efficacy, that is, in their belief, they can solve the problems and improve their lives through unified effort.”^19^, ^21^ Indeed, perceived collective efficacy beliefs emphasize how well their members work together to promote outcomes and the group's resiliency against life problems.^19^

Given the importance of head lice in girls‐aged school and their consequences, it is essential to identify factors affecting these health problems in families. Hence, the present study aimed to investigate the role of cognitive‐behavioral factors in its spread and prevention among adolescent girls.

## 2 METHODS

### 2.1 Study area

Herris (38° 14′ 50“N, 47° 6′ 59” E) is a city in the Central District of Herris County, East Azerbaijan province, the northwestern part of Iran, and serves as the capital of the county. Most citizens in Herris are overwhelmingly Azerbaijani and speak in the Turkish language, though Persian is spoken as the second language. At the 2016 census, its population was 69,093 in 20,639 households.^22^ Herris City is the abode of a total of five secondary schools that provide education solely to female students.

### 2.2 Research design and participants

The present cross‐sectional study was conducted among 276 school‐aged adolescent girls from September 2022 to January 2023 in Herris.

A multistage cluster random sample was used in female secondary schools all around this city. First of all, two out of five schools were randomly selected. Next, according to the population info, the proper sample size based on the main sample size and proportionate to the school's size was calculated and again, the people were randomly selected from all schools list.^23^ Once the students were selected to participate in the study, the parents were sent the consent form via their children. Finally, 276 parents signed (completed) the written consent form. To save time, questionnaires were completed during students' rest. The sample size was calculated based on information derived from a similar study^24^ and a confidence level of 99%, *Z* = 2.57, *SD* = 2.31, *Mean* = 14.34, 252 samples. However, since the possibility of dropping samples was taken into account, the final sample volume was larger than the calculated amount.

### 2.3 Data collection tools and scoring

To collect data, a questionnaire based on cognitive‐behavioral constructs was applied. The questionnaire recorded the following information.

Demographic variables included the father's job (Office Clerk/Laborer/Self‐employment), mother's job (Housewife/Employment), father's education (Under diploma/Diploma and higher), mother's education (Under diploma/Diploma and higher), family size (Three/Four/Six and above), hair of length (Short/Medium/Long), type of hair (Strait/Wavy/Curly), and history of lice infestation (Yes/No; Confirmation of infestation based on the health records of students).

Knowledge, perceived sensitivity, perceived severity, and barriers factors were collected using a valid and reliable questionnaire by Moshki et al. in Iran^25^; tool details are as follows.

*Knowledge* was measured using a nine‐item scale (e.g., Head lice can be transferred from animals such as cats and birds). The participants should select yes, do not know, or no for each question. For the correct answer, a score of 3, I don't know, 2, and 1 for the wrong answer were given. The higher the score, the more knowledge was concluded.

*Perceived sensitivity* (e.g., I am concerned about the possibility of getting head lice.), *perceived severity* (e.g., HLI causes a fall in students educational status.), and *barriers* (e.g., I don't have time to comb my hair in the morning when I'm in a hurry to go to school.), were rated by 20‐item scale (five items per construct), using a 5‐point Likert‐type scale ranged from 1 to 5 (*completely disagree* = 1 through *completely agree* = 5); self‐efficacy construct was measured by 5‐item scale ranged from 5 to 1 (*too much* = 5 through *very low* = 1); The better the score, the more self‐efficacy was considered.

*The parental Perceived Self‐Efficacy of HLI Prevention* scale was prepared by reviewing other questionnaires applied in similar studies.^17^, ^26^, ^27^ The validity of the tool was assessed by an expert panel (three health educationists and two epidemiologists). To assess reliability, a pilot study was conducted on 18 adolescent girls not included in the final sample. Cronbach *α* obtained 0.845. This questionnaire included seven items assessing family beliefs about their abilities: (1) to diagnose lice and nits (e.g., My family can distinguish lice from other insects); (2) to manage and treat head lice in their children (e.g., my family can treat head lice if I affected by it); (3) to get information from different sources about head lice; and (4) to plan for their child, to combing hair and bathing regularly. Participants were asked to express their perceived self‐efficacy using a scale that ranged from 0 to 4 (from strongly disagree to strongly agree).

To measure the *Head Lice Preventive Behaviors*, five questions were used (e.g., I have been using the comb several times a day for the past month.). The participants should *select always* = 3, *sometimes* = 2, and *never* = 1 for each question.^25^

### 2.4 Statistical analysis

The data were analyzed using descriptive statistics (frequency, mean, and standard deviation) and Pearson correlation coefficient test, Hierarchical linear regression. To compare group differences, independent sample *t* test was used for continuous variables; the *χ* ^2^ test was applied to compare categorical variables.

Hierarchical regression was used to determine the predictive value of demographic characteristics, knowledge, perceived sensitivity, perceived severity, barriers, and parental perceived self‐efficacy of HLI prevention for the head lice preventive behaviors in all analyses. We also used the Kolmogorov–Smirnov test for testing the normality. A significance level of *p* < 0.05 was adopted for statistical analysis using the SPSS v21.

## 3 RESULTS

### 3.1 Demographic characteristics

The demographic variable of participants is shown in Table 1. A total of 276 students participated in the study. Approximately, 9% of people (9.1%) reported infestation with head lice. A majority of participant's father's job (40.6%) were laborer and 227 (82.2%) mothers were housewife. As shown in Table 1, a statistically significant difference was found in the HLI rate by the education level of children's mothers (*p* value = 0.021). The HLI rate was significantly higher among students whose mothers were under diploma.

**Table 1:** Demographic characteristics of the subjects and its association with and no head lice.

Variable	*N* (%)	With head lice	No head lice	*p* value
		*N* (%)	*N* (%)	
Father's job				
Office clerk	93 (33.7)	5 (5.4)	88 (94.6)	0.205
Laborer	112 (40.6)	14 (12.5)	98 (87.5)	
Self‐employment	71 (25.7)	6 (8.5)	65 (91.5)	
Mothers' job				
Housewife	227 (82.2)	21 (9.3)	206 (90.7)	0.533
Employment	49 (17.8)	4 (8.2)	45 (91.8)	
Father's education				
Under diploma	101 (36.6)	12 (11.9)	89 (88.1)	0.153
Diploma and higher	175 (63.4)	13 (7.4)	162 (92.6)	
Mother's education				
Under diploma	164 (59.4)	20 (12.2)	144 (87.8)	0.021*
Diploma and higher	112 (40.6)	5 (4.5)	107 (95.5)	
Hair length				
Short	29 (10.5)	3 (10.3)	26 (89.7)	0.740
Medium	162 (58.7)	16 (9.9)	146 (90.1)	
Long	85 (30.8)	6 (7.1)	79 (92.29)	
Type of hair				
Strait	169 (61.2)	16 (9.5)	153 (90.5)	0.479
Wavy	93 (33.7)	9 (9.7)	84 (90.3)	
Curly	14 (5.1)	0 (0.0)	14 (5.6)	
Number of family members				
Three	21 (7.6)	1 (4.8)	20 (95.2)	0.571
Four	99 (35.9)	12 (12.1)	87 (87.9)	
Five	109 (39.5)	8 (7.3)	101 (92.7)	
Six and above	47 (17.0)	4 (8.5)	43 (91.5)	

* *p* value < 0.05.

### 3.2 Association between head lice and studied factors

As shown in Table 2, at the univariate level, differences based on perceived severity, perceived self‐efficacy, response efficacy, and head lice preventive behaviors were statistically significant. In other words, participants with HLI had lower levels of perceived severity, perceived self‐efficacy, response efficacy, and head lice preventive behaviors.

**Table 2:** Comparisons of cognitive‐behavioral factors among people with Head lice and no Head lice.

Variables	Status	Mean (± SD)	*p* value*
Knowledge	With head lice	15.68 (±4.33)	0.962
	No head lice	15.72 (±4.05)	
Perceived susceptibility	With head lice	11.72 (±3.65)	0.946
	No head lice	11.77 (±3.74)	
Perceived severity	With head lice	11.16 (±3.36)	0.029**
	No head lice	12.59 (±3.09)	
Perceived barriers	With head lice	31.32 (±4.69)	0.431
	No head lice	32.18 (±5.27)	
Perceived self‐efficacy	With head lice	15.44 (±4.29)	0.038**
	No head lice	17.39 (±4.48)	
Response efficacy	With head lice	14.28 (±4.39)	0.028**
	No head lice	16.43 (±5.23)	
Perceived collective family efficacy	With head lice	13.01 (±4.44)	0.078
	No head lice	14.71 (±4.94)	
Head Lice preventive behaviors	With head lice	19.56 (±3.26)	0.001**
	No head lice	21.37 (±2.12)	

* Independent samples *t* test.

** *p* value < 0.05.

The effects of demographic features and cognitive factors on head lice preventive behaviors were assessed using a hierarchical regression model (Table 3). In Step 1, demographic variables explained 3.6% of the variation in preventive behaviors (*p* value < 0.05). Table 3 shows that high protective behaviors were significantly associated with the number of family members (*ß* = 0.158; *p* value = 0.012). An additional 21.8% of the variation in preventive behaviors was explained by cognitive factors as predictor variables (Step 2) (*p* value > 0.05).

**Table 3:** Hierarchical linear regression analysis to predict head lice preventive behaviors.

Step/variables	*β* (Step 1)	*p* value	*β* (Step 2)	*p* value
*(1) Father's job*	0.059	0.345	0.012	0.833
*Mothers' job*	0.031	0.632	0.056	0.352
*Father's education*	0.082	0.231	0.060	0.340
Mother's education	0.007	0.920	0.011	0.863
Hair length	0.048	0.423	0.035	0.533
Number of family members	0.158	0.012*	0.101	0.083
*(2) Knowledge*			0.002	0.974
*Perceived susceptibility*			0.005	0.931
*Perceived severity*			0.089	0.145
Perceived barriers			0.205	0.001*
Perceived self‐efficacy			0.166	0.005*
Response efficacy			0.164	0.006*
Perceived collective family efficacy			0.290	0.001*
*R* ^2^	0.036	0.124	0.218	0.001
*R* ^2^ squared change	0.036		0.182	

* *p* value < 0.05.

As shown in Table 4, in the logistic regression analysis, perceived severity, self‐efficacy, perceived collective family efficacy, and preventive behaviors were the statistically significant predictors of HLI. Specifically, among infested individuals, the odds of perceived severity, were 1.27 (95% CI: 1.23–1.05), for perceived self‐efficacy 1.17, (95% CI: 1.03–1.32); perceived collective family efficacy 1.05 (95% CI: 1.05–1.15); and for preventive behaviors 1.32 (95% CI: 1.11–1.58) compared with those without the infestation.

**Table 4:** Logistic regression analysis to predict head lice infestation.

Variables	OR	95% CI for OR	*p* value
*Knowledge*	0.91	0.81–1.03	0.129
Perceived susceptibility	0.94	0.83–1.44	0.361
Perceived severity	1.27	1.23–1.05	0.006*
Perceived barriers	0.95	0.89–1.07	0.655
Perceived self‐efficacy	1.17	1.03–1.32	0.01*
Response efficacy	1.12	1.0–0.90	0.537
Perceived collective family efficacy	1.05	1.05–1.15	0.038*
Head lice preventive behaviors	1.32	1.11–1.58	0.002*

* *p* value < 0.05.

## 4 DISCUSSION

This study aimed to investigate the role of cognitive‐behavioral factors in its spread and prevention among adolescent girls. The findings of this study demonstrated 9.1% of students were infested with head lice. The prevalence of lice infection among schoolchildren in our study is lower compared with other communities among age‐school children, especially female students.^20^, ^28^, ^29^ This health problem may be caused by social stigma, leading to a lack of reporting and treatment, especially in adolescent girls. Therefore, head lice infection can continue to be a source of infestation for other children. On the other hand, social, cultural, and religious variables in Iran, such as using headscarves for girls in schools, contribute to the spread of HLI. Indeed, to control this condition, it is necessary to consider multiple factors.

This study found a relationship between HLI rate and the education level of children's mothers. The HLI rate was higher among students whose mothers were under diploma. Various results were seen in this finding.^30^, ^31^, ^32^, ^33^, ^34^ It may be because children's mothers with high education levels are aware of health conditions. In addition, the critical role of the Iranian mothers and the close relationship with her daughter cannot be ignored.

As the results of this research, participants with HLI had lower levels of perceived severity, perceived self‐efficacy, response efficacy, and head lice preventive behaviors (Table 2). This finding was in consist with the previous study.^5^, ^25^ This finding demonstrates that high cognitive‐behavioral factors can affect the head lice preventive behaviors and HLI rate. Therefore, training the health care providers in the school and families in terms of these factors helps reduce HLI in adolescent girls.

Based on the results, demographic variables explained 3.6% of the variation in preventive behaviors, and high protective behaviors were significantly associated with the number of family members. Additionally, 21.8% of the variation in preventive behaviors was explained by cognitive factors as predictor variables. Perceived collective family efficacy, perceived barriers, perceived self‐efficacy, and response efficacy were predictors of head lice preventive behaviors, respectively. Among all variables, perceived collective family efficacy was the strongest predictor. In the study conducted by Nezhadali et al., among mothers of school‐age children, demographic characteristics and cognitive‐behavioral factors explained 9% and 21.1% of the variation in preventive behaviors.^20^ In contradiction to our study, the research conducted by Kitvatanachai et al. reported no relationship between family size among lice‐infested and noninfested prehigh school students, but the other demographic variables were consistent with our findings.^33^ Similar results were reported by Shekarbeygi et al., which Health Belief Model constructs determined 20% of the variation in predicting pediculosis preventive behaviors (PPB), and the best determinants for PPB were perceived susceptibility, perceived barriers, and perceived self‐efficacy, respectively.^34^ Similarly, Bekry et al.,^35^ in a study among primary school‐aged children, showed that perceived susceptibility, barriers, and severity were determinants of adapting HLI‐related preventive behaviors.

Preventive cognitive‐behavioral factors can be influenced by the individual motivation to behavior, decrease its barriers, and improve perceived self‐efficacy. Raising perceived self‐efficacy and response self‐efficacy, along with promoting perceived collective family efficacy, can be effective in adopting head lice preventive behaviors. The family support received from parents/family provides children with helpful information about their ability to know how to behave as efficacious people.^19^, ^36^ If the adolescent in the school context is faced with stressful events (i.e., disease/risky condition), it can be possible adapting unhealthy behavior without family support and perceived collective family efficacy.

Perceived collective efficacy beliefs emphasize how well their members work together to promote outcomes and the group's resiliency against life problems.^19^ Considering the importance of adolescence and facing health problems at this age, it is essential to receive such a feeling from their family. In line with this fact, this research revealed that among infested individuals, the odds of perceived severity, self‐efficacy, collective family efficacy, and preventive behaviors were 73%, 83%, 95%, and 68% less, respectively, compared with those without the infestation.

According to the results, paying more attention to the role of barriers, self‐efficacy, and perceived collective family efficacy in preventing HLI in schools, which has a social stigma, is important.

First, data collection was based on self‐reporting, which is resolved by explaining the study's goal for participants. Second, the determinants of cognitive‐behavioral factors in the spread and prevention of HLI were investigated in one area of Northwest Iran, and it may be limited in generalizability to other regions and countries. Another limitation of the present study is the lack of physical examination to check for head lice in students. Only using the self‐report method to investigate the prevalence of head lice may make it difficult to generalize the results.

It is recommended that interventional programs consider cognitive‐behavioral factors to reduce the prevalence of HLI. Further, considering that part of the cognitive‐behavioral factors was identified in this study, it is suggested that future research be done using adolescent behavior study models to predict the other factors affecting HLI and preventive behaviors such as social, economic, and cultural factors. Also, the results of this study can be applied to reduce the prevalence of HLI in similar regions and cultures where the rate of infestation is high in adolescent girls in schools.

## 5 CONCLUSION

The high rate of HLI among adolescent girls in schools in Northwest Iran needs more intensive care by parents, health care providers, and teachers. Current research findings support the determinants of the cognitive‐behavioral factors in the spread and prevention of HLI. Perceived collective family efficacy, perceived barriers, perceived self‐efficacy, and response efficacy were important in predicting head lice preventive behaviors, respectively. Among all variables, perceived collective family efficacy was the strongest predictor. Given the need to reduce the HLI rate, it is necessary to involve these factors in school‐based educational programs by policymakers and healthcare providers.

## AUTHOR CONTRIBUTIONS

**Towhid Babazadeh**: Software; validation; writing—original draft. **Khalil Maleki Chollou**: Resources; writing—review and editing. **Sanaz Abedi‐Nerbin**: Visualization; writing—review and editing. **Salar Abedi‐Nerbin**: Resources; writing—review and editing. **Farzaneh Shahnavaz‐Yoshanluie**: Visualization; writing—review and editing. **Soheila Ranjbaran**: Conceptualization; supervision; writing—original draft; writing—review and editing.

## CONFLICT OF INTEREST STATEMENT

The authors declare no conflict of interest.

## ETHICS STATEMENT

This research was approved by Ethics Committee in the Sarab Faculty of Medical Sciences (Ethics Code: IR.SARAB.REC.1398.001). The supporting source had no role in the design, analysis, interpretation, or reporting of the results of this research. Written consent was obtained from the participants.

## TRANSPARENCY STATEMENT

The lead author Soheila Ranjbaran affirms that this manuscript is an honest, accurate, and transparent account of the study being reported; that no important aspects of the study have been omitted; and that any discrepancies from the study as planned (and, if relevant, registered) have been explained.

## Data Availability

All authors have read and approved the final version of the manuscript. The corresponding author had full access to all of the data in this study. The data set used for this research is available on request from the corresponding author and takes complete responsibility for the integrity of the data and the accuracy of the data analysis.
